# Uses of Glycerine

**Published:** 1865-06

**Authors:** 


### USES OF GLYCERIN.

1. Glycerin is not a fat, nor a sugar; but chemically an alcohol.

2. For use by the physician or druggist it must be absolutely *pure;* free from chlorine, lead, lime, sulphuric acid, volatile fatty acids, and glucose; and both colorless and *inodorous.* Bower's and Price's manufacture will come up this standard.*

3. Externally applied, glycerin has no specific influence over cutaneous disorders, but is an excellent emollient, especially in erysipelas, prurigo, scaly eruptions, burns and gangrenous wounds. It may be made the vehicle for any of the preparations used for skin diseases; may be diluted if necessary for open, fresh wounds, and may have bromine, carbolic acid, etc., dissolved in it for gangrene.

4. The preparation most widely approved, as a substitute for ointment, is *glyceramyl, or plasma;* composed of glycerin and starch, mixed with the aid of heat. The proportions may vary, but are usually about one part of starch to from five to eight or even sixteen parts of pure glycerin. A little glycerin added to an ordinary poultice will keep it from becoming dry and hard. A glycerole of soap is mentioned by Dr. W. A. Smith, consisting of from one to four parts of animal soap, powdered, mixed with thirty parts of glycerin, and dissolved by heating it in a water-bath.

5. Glycerin may be used as an adjuvant to the bath (twelve ounces or more at once); softening the skin pleasantly. After bathing, a few drops of it poured upon a sponge and rubbed over the body, will have a very comfortable effect.

6. Internally, glycerin is a mild laxative; in the dose of one to four drachms, it is especially available for children.

7. It can not be relied on as an *analeptic* in phthisis; but may be useful as an expectorant.

8. In pharmacy, glyceroles are more stable than aqueous, oleaginous, or saccharine preparations, while they are less

* The best that I have been able to obtain from any other source but those above mentioned, has a perceptible and somewhat disagreeable odor when rubbed upon the hand; besides giving *copious* precipitates with solution of nitrate of silver, and with chloride of barium; which do not at all affect strictly pure glycerin.

stimulant, and generally more agreeable than alcoholic tinctures. Glycerole of quinine, of iodo-quinine, of iodide of iron, of subnitrate of bismuth, of lactucarium, of atropia, and of acetate of lead have been considerably used. Among many others that have been suggested, that of ipecac, is particularly desirable on account of the great fermentability of the ordinary syrup. I would add, also, especially the following:—

*Glycerole of Quinia with Strychnia.*—℞. Strychniæ, gr. j. Quin. Sulphat., ℈ij. Glycerin, f℥iv M. ft. sol. Dose, a fiuid drachm.

A combination of nux vomica with quinine has recently been found valuable in the cure of obstinate chronic intermittents, which resisted quinine alone. The above preparation will answer for the same purpose.

*Glycerole of Ammonio-Citrate of Iron with Quinine.*—℞. Ammonio-ferri et quiniæ citrat., ℈ij. Glycerin, f℥iv. M. ft. sol. Dose, one or two fluid-drachms.

The addition of this to cod-liver oil destroys the nauseousness of its taste and effect; especially with the further addition of one or two drops of oil of cloves to each ounce of the mixture.

*Glycerole of the Sulphites of Soda and Magnesia.*—Worthy of careful and extensive trial as an antizymotic; upon the idea of Polli (*Am. Jour. of Med. Sciences*, April, 1863, p. 467, and Jan., 1865);* in diphtheria, etc.

*Glycerole of Phosphate of Iron.*—℞. Ferri phosphat., ʒj. Glycerin, f℥iv M. ft. Dose, two fluid-drachms.

This requires slight shaking when given, although nearly the whole is dissolved.

*Glycerole of Oxide of Zinc.*—℞. Zinci oxid., ℥ss. Glycerin, f℥iv M. S. Shake before using. For external application.

I advise this as an emollient for severe burns, and in eczema, herpes, pemphigus, etc.

*Glycerole of White Lead.*—℞. Plumb. carbonat., ʒj. Glycerin, f℥iv M.

This is an almost perfect solution, all of the carbonate being suspended on slight agitation. It is recommended to be applied

* *Vide* Dr. W. F. Atlee's account, in that journal (Jan., 1865, p. 82), of the good effects of sulphite of soda in pyæmia; and M. Carey Lea's paper, in the same number (p. 84), on the Transformation of Sulphites in the system.

to the edges of the lids with a hair pencil, in conjunctivitis, or general ophthalmia, acute or chronic; to inflamed hemorrhoids, and to the surface over bones affected with periostitis, of scrofulous or other origin. In the last-named affection I have found carbonate of lead of remarkable utility; and glycerin will introduce it more readily than any unguent.

Many other applications of the same or other similar preparations might be suggested. I can not pretend to exhaust the subject. Yet, in the American Pharmacopœia, but a few lines are given to glycerin, and none to any of its preparations; and in the British Pharmacopœia, it is merely mentioned as a solvent for tannin in *supposit. tannic.* It can hardly be much longer left to so insignificant a share of officinal recognition.—*Med. and Surg. Rep.*

